# Nanomechanics of Retained Austenite in Medium-Carbon Low-Temperature Bainitic Steel: A Critical Analysis of a One-Step versus a Two-Step Treatment

**DOI:** 10.3390/ma15175996

**Published:** 2022-08-30

**Authors:** Songbo Zhou, Feng Hu, Kun Wang, Chengyang Hu, Wen Zhou, Serhii Yershov, Kaiming Wu, Zhicheng Zhang, Xianming Pan

**Affiliations:** 1 The State Key Laboratory of Refractories and Metallurgy, Wuhan University of Science and Technology, Wuhan 430081, China; 2 Collaborative Innovation Center for Advanced Steels, Wuhan University of Science and Technology, Wuhan 430081, China; 3 Metals Valley & Band (Foshan) Metallic Composite Co., Ltd., Foshan 528000, China; 4 Daye Special Steel Co., Ltd., Hubei Province Key Laboratory of High Performance Special Steel, Huangshi 435001, China

**Keywords:** bainitic steel, two-step, retained austenite, nanoindentation

## Abstract

A two-step bainitic treatment with a final isothermal temperature below M_S_ was adopted to obtain bainitic steel with abundant retained austenite (RA). Nanoindentation testing was used to investigate the stability of RA in bainite steel and clarify the effect of RA on the deformation of medium-carbon steel. The results showed that, in contrast to the traditional one-step approach, a greater amount of nanoscale RA film was obtained using the two-step treatment. This was due to a lower final bainitic transformation temperature, which induced a higher carbon concentration in the untransformed austenite in the stasis stage; this resulted in untransformed austenite with a higher carbon content existing as RA rather than forming martensite in the subsequent cooling process. In addition, it was determined that the increased stability of RA during the two-step transformation delayed the pop-in point.

## 1. Introduction

A bainitic transformation at an intermediate temperature (usually 300–500 °C) is applied in medium-carbon (0.2–0.3 wt.%) high silicon (~1.50 wt.%) steel [1,2,3,4] to obtain a submicron bainitic ferrite (BF) lath (100–200 nm), carbon-rich retained austenite (RA), and an appropriate amount of blocky martensite. The resulting steel has an impact energy of approximately 24–44 J and an impact strength of almost 1600–1800 MPa. A few researchers [1,2] have shown that the impact toughness is mainly due to the deformation-induced martensitic transformation of metastable RA and the resultant transformation-induced plasticity (TRIP) effect during the strain process. However, the formation of blocky martensite itself has no obvious effect on the toughness during water-quenching after the isothermal bainitic process, implying that there is an optimum amount of RA required to increase the impact toughness of bainitic steels.

It has been reported [5,6,7] that an increase in the volume fraction of nano- or submicron-scale RA increases the impact toughness of bainitic steels. Two-step [8] and multi-step [9] treatments for bainitic transformations have been used to improve toughness where newly formed nano-sized bainitic ferrite (BF) in untransformed austenite and blocky M/RA were significantly refined and decreased. It has been observed that RA with the best impact behavior is as a film between subunits of bainite, rather than as blocky regions between sheaves of bainitic ferrite [6]. However, there are few studies on the micro/nanoscale deformation of RA in bainitic steel.

Nanoindentation is advanced microscale (submicron/nanometer) mechanical measurement technology that can be used to investigate the deformation mechanism of microstructures under a compressive stress. Furnemont et al. [10] and He et al. [11] studied the nanohardness of isothermally transformed bainite. However, these studies focused on the different morphologies of bainite rather than its constituent phases. Thus, the results were also considered to represent the bulk nanohardness for different bainite morphologies. Fujita et al. [12] showed that coarse MA islands (larger than 5 µm) trigger strain localization whereas finely dispersed MA islands can contribute to the improvement of the strain capacity of bainitic steels. This implies that exploring the property of each constituent of bainite microstructures is also important to understand the nature of the overall mechanical behavior. Misra et al. [13] and He et al. [14] used nanoindentation testing and transmission electron microscopy with electron backscatter diffraction (TEM/EBSD) to study the microdeformation of RA in quenched-and-partitioned (Q-P) martensitic steel. These studies found that different microstructures had different microdeformations. Thus, as with Q-P martensitic steel, it is supposed that phases with different morphologies may have a significant impact on microdeformation in bainitic steel; this requires further in-depth studies. In this study, bainite steel containing RA was prepared using the concept of a two-step bainite treatment. Due to the small scale and diversified morphology of metastable austenite, it is difficult to use conventional methods for a deformation analysis. The stability of RA in bainite steel was deeply understood by a nanoindentation test and the effect of RA on the deformation of medium-carbon steel was clarified. As the incipient plasticity behavior of bainitic steel is not known, an insight into nanoscale deformation mechanisms could provide the pathway to establish a relationship between local deformation characteristics and the overall deformation behavior. Revealing the inherent deformability of bainitic steel and establishing a connection between the deformation mechanisms across a length scale can enable the prediction of deformation behavior.

## 2. Experimental Procedures

Table 1 shows the measured chemical composition of the investigated steel. The experimental steel was made in a 50 kg vacuum medium-frequency induction smelting furnace. A steel plate with a thickness of 20 mm was hot rolled by a small two-roll reversible rolling mill. The specimens were austenitized at 1000 °C for 30 min; the one-step (isothermally treated at 350 °C for 4 h in a box furnace and finally air-cooled to room temperature) and two-step (isothermally treated at 300 °C for 2 h in a box furnace, then at 250 °C for 24 h in another salt bath furnace, and finally air-cooled to room temperature) treatments for the bainitic transformation were then performed for a comparison.

Optical microscopy (OM; DM2700 M, Weztlar, Germany), scanning electron microscopy (SEM; Thermo Fisher Apreo S HiVac, Wyman Street, Waltham, MA, USA), and electron backscattered diffraction (EBSD; Symmetry EBSD, Oxford, UK) were used to examine the microstructures and to determine the distribution, size, and morphology of RA, bainitic ferrite, and martensite. The specimens were prepared by taking 10 mm cross-sections of the treated steels. The specimens were ground and polished using standard techniques and etched in a 4 vol% nital solution.

The transmission electron microscopy (TEM; JEM-F200, Kyoto, Japan) specimens were prepared by machining the treated steels into 3 mm diameter rods and then slicing these rods into discs with a thickness of 100 µm. The discs were ground down to a thickness of 50 µm using 2000 grit SiC paper and then electro-polished in an electrolyte consisting of 5 vol.% perchloric acid, 15 vol.% glycerol, and 80 vol.% methanol at 50 V using a twin-jet unit.

The X-ray diffractometry (XRD; Xpert Pro MPD; operated at 40 kV and 45 mA with Cu Kα radiation) specimens were ground, polished, and slightly etched in 4 vol% nital for the phase characterization. The 2θ scanning angles varied from 40° to 110° with a stepping angle of 0.033°. Finally, the volume fraction of RA was calculated by measuring the integrated intensities of the (111), (200), (220), and (311) austenite peaks and comparing them with the (110), (200), (211), and (202) martensite peaks [15].

Nanoindentation experiments were carried out on a nanoindentation tester (Hysitron TI 950 TriboIndenter) with a Berkovich tip. The nanoscale deformation experiments were conducted using a load-controlled mode at a loading rate of 100 μN s^−1^ with the maximum load set to 2000 μN. Here, the objective was to observe any differences in the load-displacement plots that could provide an insight into the deformation mechanism. A post mortem EBSD study of the indented samples was carried out at a step size of 50 nm to explore the grain orientation in the plastic zone surrounding the indented region [16]. The specific method has been described in detail in preliminary research work [17].

## 3. Results

### 3.1. Microstructure

**Figure 1:**
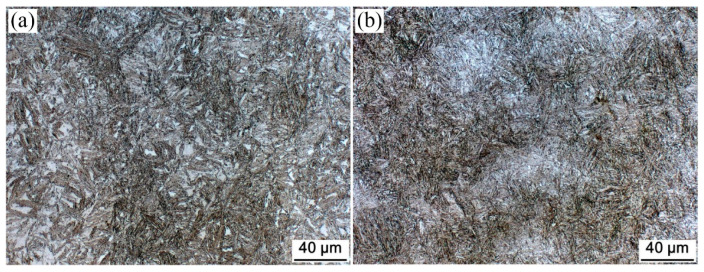
OM micrographs of the investigated steel for (**a**) one-step and (**b**) two-step treatments.

**Figure 2:**
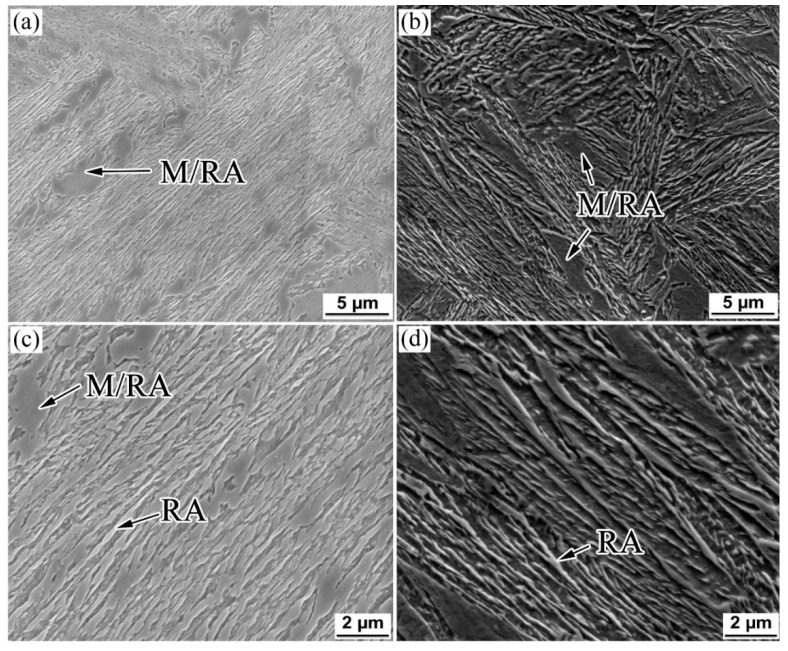
SEM micrographs of the investigated steel for (**a**,**c**) one-step and (**b**,**d**) two-step treatments.

**Figure 3:**
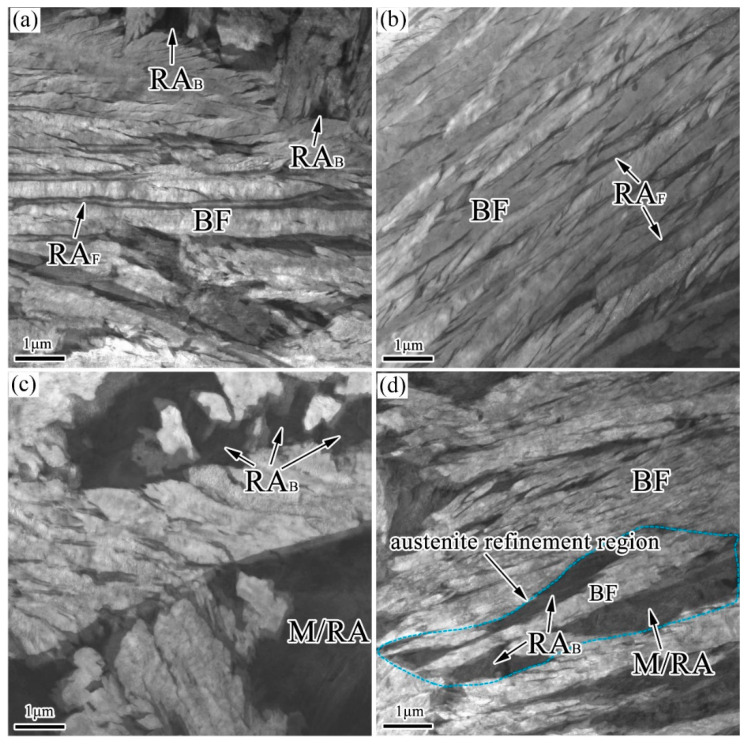
TEM micrographs of the investigated steel for (**a**,**c**) one-step and (**b**,**d**) two-step treatments.

Figure 1, Figure 2 and Figure 3 show the OM, SEM, and TEM micrographs of the different specimens after the bainitic transformation. As shown in Figure 1, the microstructure consisted of white areas of blocky martensite/retained austenite (M/RA) and brown bainitic ferrite (BF) zones. Compared with the samples prepared using the one-step method, the BF transition of the samples prepared using the two-step method was higher and the fraction of the white area was reduced (Figure 1b). The microstructure of the samples prepared using the one-step method consisted of a bainite sheaf, microblocky M/RA, and a retained austenite film (RA_F_) (Figure 2a,c and Figure 3a,c). After the two-step treatment, the size of blocky M/RA and RA was refined (Figure 2b). In addition, the volume fraction of blocky M/RA significantly decreased (Figure 2b). In the TEM micrographs of the two-step transformation specimens, we observed that the microstructure of the bainite sheaf was composed of a bainite ferrite lath and nanofilm RA (Figure 3b). Two forms of retained austenite (microblocky RA and nanofilm RA) were observed by SEM and TEM, in which microblocky RA was located between the bainite sheaf and nanofilm RA was located between the BF lath. The prior austenite in the light blue region of Figure 3d was refined to RA_B_ and M/RA with the transformation of the bainite in the two-step method.

### 3.2. Nanoindentation Tests

**Figure 4:**
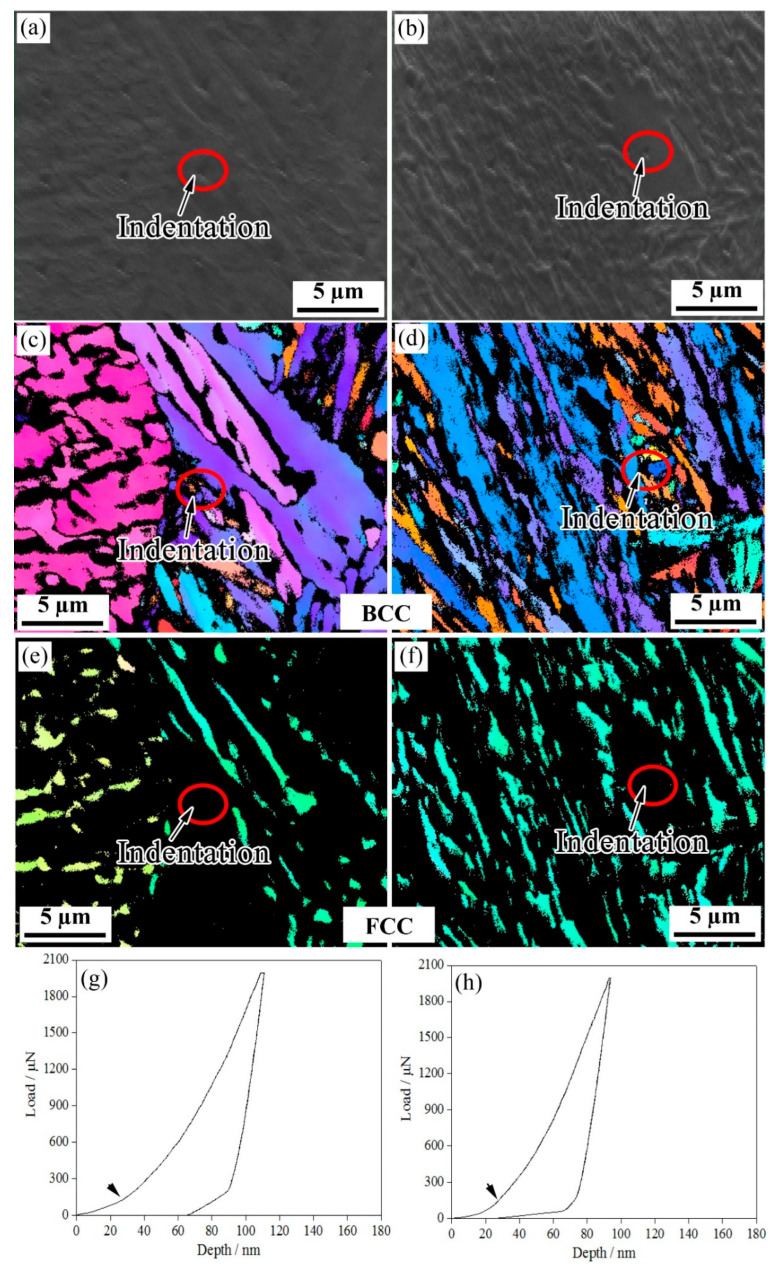
Nanoindentation of blocky M/RA in the investigated steel for (**a**,**c**,**e**,**g**) one-step and (**b**,**d**,**f**,**h**) two-step techniques. (**a**,**b**) SEM micrographs, (**c**,**d**) BCC structures, (**e**,**f**) FCC structures, and (**g**,**h**) representative load–displacement plots.

**Figure 5:**
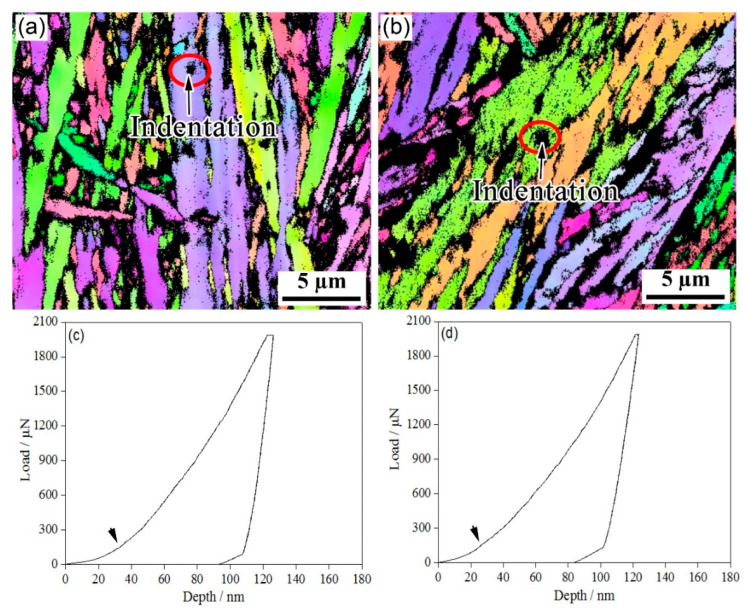
Nanoindentation of bainite sheaf in the investigated steel for (**a**,**c**) one-step and (**b**,**d**) two-step techniques. (**a**,**b**) BCC structures and (**c**,**d**) representative load–displacement plots.

**Figure 6:**
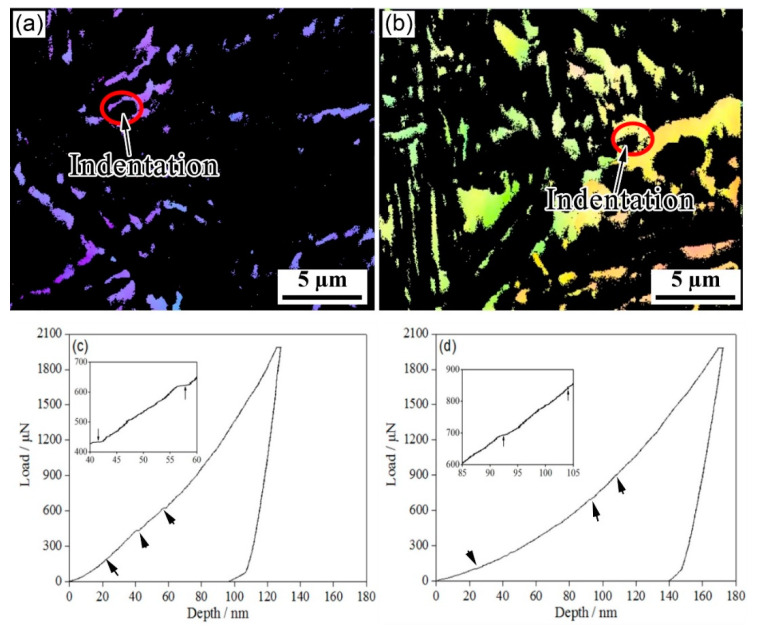
Nanoindentation of blocky retained austenite in the investigated steel for (**a**,**c**) one-step and (**b**,**d**) two-step techniques. (**a**,**b**) FCC structures and (**c**,**d**) representative load–displacement plots.

**Figure 7:**
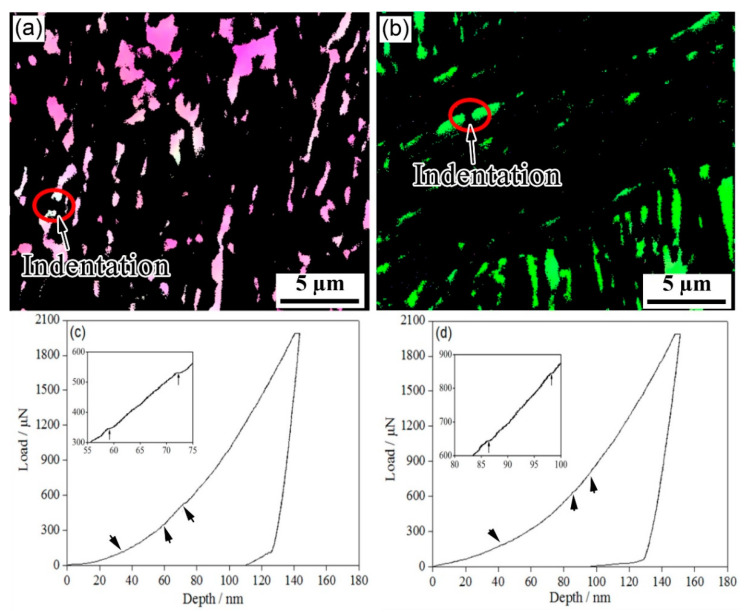
Nanoindentation of RA film in the investigated steel for (**a**,**c**) one-step and (**b**,**d**) two-step techniques. (**a**,**b**) FCC structures and (**c**,**d**) representative load–displacement plots.

Figure 4, Figure 5, Figure 6 and Figure 7 show the EBSD characterization of the deformation under a nanoindentation of the microstructures of the steels treated using the two processes. Figure 4 and Figure 5 show the nanoindentation–EBSD characterization of the blocky M/RA and BF, respectively. For the samples processed using the one-step method, the indentation depths of the blocky M/RA and BF lath were about 110 nm and 130 nm, respectively; the two-step blocky M/RA and BF had a smaller indentation depth of about 95 nm and 125 nm, respectively. Under the same heat treatment conditions, and compared with BF, all load-depth (L-D) curves of the blocky M/RA components had a larger loading slope and a shallower penetration depth. Kadkhodapour et al. [18] also found a higher nanohardness near martensite, which is caused by geometrically necessary dislocations. The pop-in points in the L-D curves of M/RA and BF were activated by pre-existing dislocations or dislocation nucleation. In particular, dislocation nucleation leads to a pronounced pop-in with a sudden displacement burst, resulting in the first pop-in [19].

Figure 6 shows the nanoindentation–EBSD characterization of blocky RA. A number of minor pop-in points (Figure 6c,d) were observed in the L-D curves of blocky RA; these were related to both the nucleation of the dislocations and the martensitic transformation of RA under stress [20]. In the one-step method, the indentation depths of the pop-in points related to the phase transition were between 40 nm and 80 nm; in the two-step method, the indentation depth of the pop-in was over 80 nm. The two-step method delayed the pop-in point. At the same time, as martensite is much harder than austenite, and the lattice parameters of martensite are larger than those of austenite, the martensitic transformation of RA during the deformation inevitably increased the strain hardening rate.

As the phase transformation stability of the RA film was more pronounced, EBSD was used to characterize the RA phase transformation stability of the film under nanoindentation (Figure 7). An analysis of the L-D curve showed that there were two pop-in points in the one-step method after the first pop-in, with a load range of 300–600 μN; the two-step method increased to more than 600 μN. These pop-in phenomena are said to be related to the transformation of RA into martensite [10,20]. As martensite is much harder than austenite, and the lattice parameter of martensite is larger than that of austenite, the appearance of martensite during the deformation must have increased the strain hardening rate. Due to the extended isothermal time in the two-step method, a large quantity of bainite formed at 300 °C and then was isothermally maintained at 250 °C. Several of the carbon atoms in the bainite lath were lost to capture the defects and to distribute them to the austenite, making the retained austenite film richer in carbon. The two-step isothermal method could further refine the blocky RA, change the morphology of the blocky RA and the distribution of carbon, and improve the stability of the retained austenite. From these considerations, we inferred that the blocky RA contributed to greater work hardening and strengthening at the initial stage of deformation whereas the RA film prolonged uniform elongation in the high-strain region [21].

Table 2 shows the nanohardness of the different phases in the specimens after the bainitic transformation, for which 20 datasets were analyzed. The nanohardness of blocky M/RA and BF for one-step bainite were 8.45 ± 1.15 GPa and 5.20 ± 0.40 GPa, respectively. The nanohardnesses of the blocky (2.80 ± 0.30 GPa) and film (3.15 ± 0.45 GPa) RA were much lower than those of the blocky martensite and bainite sheaf; the nanohardness of the RA film was greater than that of blocky RA. Compared with one-step bainite, two-step bainite had higher nanohardnesses of blocky M/RA and BF (about 8.25 ± 1.55 GPa and 5.85 ± 0.45 GPa, respectively), but lower nanohardnesses of blocky and film RA (about 3.25 ± 0.25 GPa and 3.75 ± 0.35 GPa, respectively).

### 3.3. Mechanical Properties

Table 3 shows the mechanical properties of the different specimens after the bainitic transformation. The tensile strength (1560 MPa) of two-step bainite was significantly higher than that of one-step bainite (1345 MPa), but the elongation (12.5%) was lower than one-step bainite (16.0%). The impact energy (46 J) of two-step bainite was higher than that of one-step bainite (24 J). Meanwhile, the HV1 hardness (498) of the two-step method was significantly higher than that of the one-step method (415).

## 4. Discussion

### 4.1. Transformation Kinetics

**Figure 8:**
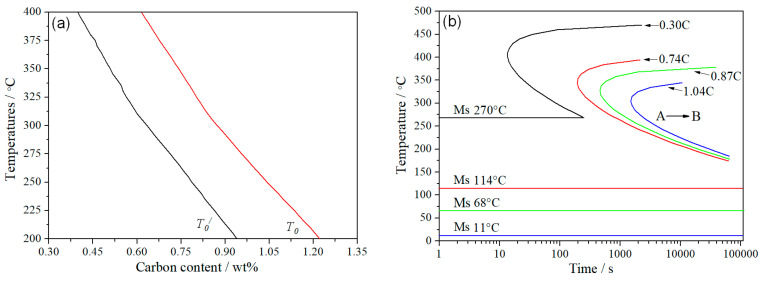
Calculations for (**a**) equilibrium transformation curve and (**b**) TTT isothermal bainitic transformation diagram (T_0′_: equilibrium transformation temperature in strain energy; A*_e_*_3_: para-equilibrium transformation temperature; A → B: austenite transformed to bainite).

Figure 8 shows the T_0_ (equilibrium transformation) curve calculated by MUCG83 software [22]; the isothermal bainitic transformation diagram was calculated by J-MatPro 4.0 software (Cambridge, UK) [23]. The carbon was diffused from supersaturated bainite to an untransformed austenite phase in the isothermal process. When the carbon content in the austenite reached a temperature of T_0_ or T_0′_, the bainitic transformation process almost stopped, causing an incomplete evolution in the bainitic steel [24]. The carbon content of austenite is, theoretically, 0.74 wt.% and 0.87 wt.% after isothermal bainitic transformations at 350 °C and 300 °C, respectively.

**Figure 9:**
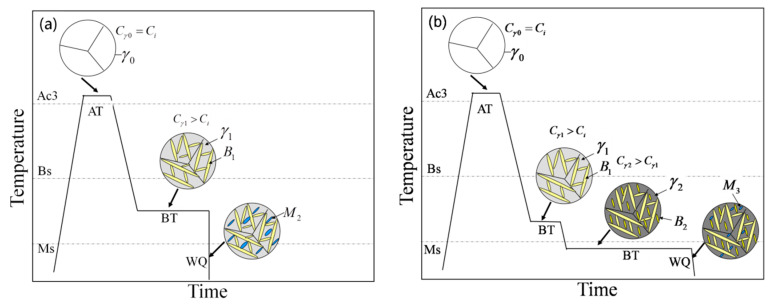
Schematic diagram of bainite transformation: (**a**) one-step and (**b**) two-step techniques (A_c3_: temperature of ferrite completely transformed into austenite during heating; Bs: starting transformation temperature of bainite; M_S_: starting transformation temperature of martensite; AT: austenitizing temperature; BT: bainitic transformation temperature; *WQ*: water-quenching; *γ*_0_: austenite microstructure in austenitizing process; *γ*_1_: austenite microstructure in one-step bainitic transformation process; *γ*_2_: austenite microstructure in two-step bainitic transformation process; *B*_1_: bainite microstructure in one-step bainitic transformation process; *B*_2_: bainite microstructure in two-step bainitic transformation process; *M*_2_: martensite microstructure in water-quenching process for one-step bainitic transformation; *M*_3_: martensite microstructure in water-quenching process for two-step bainitic transformation; C_i_: carbon content of alloy elements; C_γ0_: carbon content of austenite microstructure in austenitizing process; C_γ1_: carbon content of austenite microstructure in one-step bainitic transformation process; C_γ2_: carbon content of austenite microstructure in two-step bainitic transformation process).

The isothermal bainitic transformation temperature was 300–350 °C, which was between the B_S_ and M_S_ (bainitic start and martensitic start transformation temperatures) (Figure 9a). In this experiment, the microstructure of the one-step bainitic transformation was BF, RA, and an amount of martensite. The reason was that during the bainitic transformation, the carbon content of the untransformed austenite was about 0.74 wt.% after the isothermal transformation at 350 °C in one-step bainite. During the final quenching process, most of the untransformed austenite further transformed into martensite; due to this, we predicted that the M_S_ could be reduced to 114 °C, which is much higher than room temperature (Figure 8b).

At the same time, the carbon content of the untransformed austenite was about 0.87 wt.% after an isothermal transformation at 300 °C; we predicted that the M_S_ could be reduced to 68 °C (Figure 8b). The untransformed austenite further transformed into new BF in the second bainitic transformation process because the M_S_ was actually lower than 250 °C, which caused carbon enrichment in the untransformed austenite (the carbon content of austenite is, theoretically, 1.04 wt.% after an isothermal bainitic transformation at 250 °C). During the final quenching process, a small amount of the untransformed austenite further transformed into martensite; due to this, we predicted that the M_S_ could be reduced to 11 °C, which is slightly lower than room temperature. Therefore, the two-step treatments could further promote a bainitic transformation, not only increasing the contents of the BF and RA film, but also significantly reducing the amount and refining the size of blocky M/RA (Figure 9b).

### 4.2. Deformation of Retained Austenite

Medium-carbon bainite is a new type of steel with an ultra-high strength, high plasticity, and toughness. These excellent properties are attributed to the reasonable ratio of BF to RA [14]. The ultra-high strength comes from a submicron-sized BF lath and the solid solution strengthening of alloy elements; the toughness depends on the stability of RA [15]. There was a substantial amount of martensite in the one-step bainitic transformation process where the hard-brittle martensite did not effectively absorb energy and passivate cracks and had no obvious effect on the impact toughness of the steel. In contrast, there was only a small amount of martensite in the two-step bainite transformation process and the content of RA was also higher than that of the one-step bainite transformation process. A plastic martensitic transformation [6] occurred under a stress–strain state because the stable RA contained a greater number of potential nucleation particles that transformed into martensite during the effect of external stress. Under this condition, strain hardening occurred when the volume of RA increased, which induced martensite nucleation and the transformation to martensite, thus improving the local hardness. This plastic martensitic transformation could effectively alleviate the local stress concentration, delay crack formations, and prevent crack propagation, which effectively improved the overall deformation ability of the structure and thus delayed the occurrence of necking by the TRIP effect [25,26].

Studies of L-D curve behaviors by nanoindentation in austenite stainless [27] and Q&P martensite [8] steels have shown that the first pop-in in the austenite phase is most likely to be a consequence of the nucleation of dislocations; the second and subsequent pop-ins in the austenite phase are attributed to indentation-induced twin-type martensite [28]. At the same time, a greater indentation depth of the pop-ins indicates that RA is more stable. The results of Figure 6 and Figure 7 show that the RA film was more stable than blocky RA and the stability of RA in two-step bainite was higher than that of one-step bainite. During nanoindentation, the test was initiated without the formation of large pop-ins. The mechanical behavior of austenite during elastic–plastic transformation is closely related to its morphology and substructure [29]. In the one-step method, blocky austenite with a low carbon content had a low stability. After the isothermal bainite transformation, it was partially transformed into martensite during cooling, thus forming blocky M/RA. Due to the poor stability of blocky austenite and its less obvious TRIP effect, blocky M/RA is not conducive to ductility and toughness [30,31].

## 5. Conclusions

To explore the deformation mechanism of retained austenite with different morphologies under microstress, nanoindentation combined with EBSD was used to characterize the phases of steels treated using one-step and two-step processes. The main conclusions can be summarized as follows:

(1) Compared with the one-step isothermal transformation, the two-step isothermal transformation led to the size of the blocky M/RA being significantly reduced and refined and to an amount of austenite continuing to transform into a fine BF lath. Compared with blocky RA, thin-film RA had a higher surface-to-volume ratio; this made the interface larger, but the diffusion path shorter.
(2) An EBSD analysis and nanoindentation tests showed that the RA film was more stable than blocky RA. The low mechanical stability of blocky RA was due to various defects that acted as nucleation sites of the martensitic transformation. Compared with the one-step treatment, the increase in stability of RA under the two-step transformation delayed the occurrence of the pop-in points. The nanohardness was also significantly improved.

## Figures and Tables

**Table 1:** Chemical composition of the investigated steel (wt.%).

C	Si	Mn	Cr + Mo + Ni + Cu	Ti + Nb
0.30	1.46	1.97	3.00	0.04

**Table 2:** Nanohardnesses of the different phases for the investigated steel (GPa).

Phase	One-Step			Two-Step		
	Min	Max	Ave	Min	Max	Ave
Blocky M/RA	8.18	10.92	8.45 ± 1.15	8.62	12.14	8.25 ± 1.55
BF	4.68	5.76	5.20 ± 0.40	5.14	6.42	5.85 ± 0.45
Blocky RA	2.68	3.64	2.80 ± 0.30	2.62	3.78	3.25 ± 0.25
RA Film	2.76	4.38	3.15 ± 0.45	3.36	4.34	3.75 ± 0.35

Note: the nano hardness measurement of each phase was based on a statistical analysis of 20 groups of load–displacement curves and the average value was taken.

**Table 3:** Mechanical properties of the investigated steel.

Specimens	Tensile Property		Impact Property at 20 °C		HV1
	R_m_, MPa	A, %	Impact Energy, J	Average, J	
One-step	1345	16.0	23, 23, 26	24	415
Two-step	1560	12.5	43, 50, 46	46	498

## Data Availability

Not applicable.
